# Cycling: de Hartog et al. Respond

**DOI:** 10.1289/ehp.1003227R

**Published:** 2011-03

**Authors:** Jeroen Johan de Hartog, Hanna Boogaard, Gerard Hoek, Hans Nijland

**Affiliations:** University of Utrecht, Institute for Risk Assessment Sciences, Utrecht, the Netherlands, E-mail: j.j.dehartog@uu.nl; Netherlands Environmental, Assessment Agency, Bilthoven, the Netherlands

We thank Int Panis for his thoughtful comments on our article (de Hartog et al. 2010), and we broadly agree with his comments. In fact, we discussed most of the issues—including the limitation to impact on mortality, sensitive subgroups, route choice, and activity substitution—in our paper.

The first issue discussed by Int Panis is whether we underestimated the difference in minute ventilation between cyclists and car drivers; however, his comment was based on a recent Belgian study (Int Panis et al. 2010) that was not published at the time of our study. In our analysis we used a ratio of 2.2 [the average of two Dutch studies that closely agreed (van Wijnen et al. 1995; Zuurbier et al. 2009)], whereas the Belgian study (Int Panis et al. 2010) found a ratio of 4.3. The difference is probably explained in part by differences in cycling speed: 12 km/hr in the recent Dutch study (Zuurbier et al. 2009) and > 19 km/hr in the Belgian study (Int Panis et al. 2010). In urban areas, the average cycling speed is about 15 km/hr, including stop time. Rather than replacing the previous estimates by with the newer Belgian estimate, we believe that the best current estimate would be the average of the ratios of the three available studies. This would lead to a ratio of 2.9. Use of this ratio based on more studies clearly would not tip the balance between cycling and car driving as Int Panis suggests. We think it is stretching the data too much to use deposited particle mass (actually 5.9–8.99 higher in the Belgian study) for the analysis, because the long-term epidemiological studies we used are based on concentrations measured in outdoor air. In the most likely estimate we provided for air pollution [based on black smoke, which better represents traffic exposures than PM_2.5_ (particulate matter < 2.5 μm in aerodynamic diameter)], even including these estimates would not make a difference.

As we noted in the “Discussion” of our article (de Hartog et al. 2010), cyclists have more opportunity in urban areas to choose low-exposure routes. This would indeed result in smaller differences in inhaled doses between cyclists and car drivers than we used. We agree that we may have overestimated the air pollution risks related to cycling because, in general, subjects who cycle are healthier than those who respond in long-term epidemiological studies. However, with increasing evidence that air pollution—through oxidative stress and inflammation—may also increase preclinical cardiovascular disease, including atherosclerosis (Brook et al. 2010), long-term effects of air pollution are not limited to mortality in the most sensitive subjects.

Another issue deals with the large number of nonfatal bicycle accidents reported in a recent assessment in Belgium (Aertsens et al. 2010). We do not want to downplay the importance of these accidents; however, because Aertsens et al. (2010) exclusively reported accidents in cyclists, the study cannot be used in a comparative assessment of the risks of cyclists and car drivers, the topic of our paper. There is therefore no basis for Int Panis’s statement that inclusion of this information could easily have tipped the balance between risks and benefits. As we discussed in our article (de Hartog et al. 2010), we also did not take into account the benefits of physical activity on quality of life and other nonfatal health effects. We welcome an attempt to systematically make this assessment. In an assessment of traffic accident risks and benefits from air pollution and physical activity in the general population using disability-adjusted life years (DALYs), Woodcock et al. (2009) found that about about 80% of the calculated DALYs of all stressors were due to loss in life years, so we do not expect our conclusion to be much affected when morbidity is assessed. Finally, we fully agree with the statements about the importance of reducing risks from accidents and air pollution for commuters. Our paper should not be interpreted as a plea for ignoring these important risks.
